# Increased Prevalence of Fragmented QRS in Randomly Selected Group of Multiple Myeloma Patients: A Pilot Study to Assess Prevalence

**DOI:** 10.7759/cureus.20635

**Published:** 2021-12-23

**Authors:** Angel López-Candales, Fuad Habash, Srikanth Vallurupalli

**Affiliations:** 1 Cardiovascular Medicine, University of Missouri - Kansas City, Kansas City, USA; 2 Cardiology, Baylor College of Medicine, Houston, USA; 3 Cardiovascular Disease, University of Arkansas for Medical Sciences, Little Rock, USA

**Keywords:** cancer, multiple myeloma, onco-cardiology, chemotherapy associated cardiotoxicity, fragmented qrs, electrocardiography (ecg)

## Abstract

The presence of fragmented QRS (fQRS) on surface electrocardiograms (ECGs) has been associated with the myocardial scar in certain cardiac conditions and has been proposed as a potential marker of adverse cardiac outcomes. Since myocardial fibrosis not only has been recognized as a side effect of certain cancer therapies but also, fQRS have been shown in some breast cancer and lymphoma survivors post-chemotherapy treatment, we decided to investigate if fQRS could also be seen in multiple myeloma (MM) patients since this association has never been described. For this pilot study, we analyzed ECGs from 137 randomly selected MM patients during different stages of their treatment, and fRQS was found in 42% of these cases. The prevalence was much higher than the reported prevalence for the general population (19.0%) but closer to that reported for patients with myocardial infarction (39.5%). We also found that female MM patients are more commonly affected than women in the general population. Based on this small random sampling analysis, fQRS appears highly prevalent among unselected MM patients. This novel finding of fQRS in MM patients certainly adds to the growing data of cases among different cancer patients, opening the door to conduct larger prospective studies that undoubtedly will help to create a more robust database regarding the potential utility of this ECG abnormality.

## Introduction

Fragmentation of the QRS (fQRS) from surface electrocardiograms (ECG) is a term that has been recently used to characterize subtle conduction abnormalities that include abnormal slurrings, notching, or aberrancies seen within QRS complexes. The presence of these fQRS was initially described in surface ECGs of individuals with left ventricular hypertrophy and in patients with myocardial scar after a myocardial infarction [1,2].

However, fQRS was later identified as a marker of depolarization abnormality and was consequently considered a potential noninvasive marker for identifying patients at risk of sudden cardiac death [3]. This conceptualization was derived from the notion that alteration of electrical signals traversing the myocardium will distort QRS morphology because of the resultant inhomogeneous activation that will occur as electrical signals travel across the myocardium and their conduction is altered by interspersed myocardial scarring within the cardiac architecture [3]. Therefore, these findings changed the prior notion that fQRS was no longer specific for coronary disease since it could be linked to other cardiac conditions such as nonischemic cardiomyopathy, congenital heart disease, arrhythmogenic right ventricular dysplasia cardiomyopathy, and the Brugada syndrome that were also characterized by some pathological process resulting in myocardial fibrosis [4-8].

Follow up studies that have now included an additional population of patients, including the Coronary Heart Disease Study of the Finnish Mobile Clinic Health Examination Survey, Innovation to Reduce Cardiovascular Complications of Diabetes at the Intersection (ARTEMIS) study, and the Finnish Genetic Study for Arrhythmic Events (FinGesture) study, which collects clinical and autopsy data from victims of sudden cardiac death, have shown that the overall prevalence of fQRS in the general population is 19.0%. In comparison, patients with suspected coronary artery disease (CAD) have a prevalence of 22.3%, a number that significantly increased to 35.3% for those with known CAD and is 39.5% in those patients with a prior myocardial infarction. However, the prevalence of finding fQRS increases significantly to 53.8% in those patients dying from sudden cardiac death [9].

In the case of women, all these studies did show a significantly lower prevalence of fQRS than that reported for men regardless of the population studied [9].

Since myocardial fibrosis has been a well-recognized side effect of cancer therapy, the search for the presence of fQRS among cancer patients was a logical extension to assess if this depolarization abnormality was found. Since breast cancer is the most prevalent of all cancers [8], this was the first malignancy studied. Data published by Adar and associates was the first to demonstrate that breast cancer patients treated with radiotherapy has evidence of fQRS on the surface ECG [10]. This was later confirmed, demonstrating that fQRS was also found among breast cancer patients receiving chemotherapy [10]. Of note, these ECG abnormalities were not only noted before the onset of any symptoms but, most importantly, before any echocardiographic abnormalities were documented [11].

The presence of fQRS was later identified among 40.8% of non-Hodgkin lymphoma patients treated with R-CHOP regimens [12]. Based on these findings, the investigators conducting this study proposed that the development of the fQRS pattern in response to cancer therapy might be considered as a potential new tool for the noninvasive assessment of potentially related chemotherapy-induced cardiotoxicities [12].

Although multiple myeloma (MM) is still considered a rare disease, it accounts for 1% of all cancers [13,14]. However, it is the second most common hematologic malignancy after lymphoma [13,14]. MM is not only a unique malignancy that covers a wide spectrum of clinical presentations ranging from plasma cell dyscrasias to overt plasma cell leukemia and extramedullary myeloma but is also characterized by recurrent relapsing courses [15]. The current approved treatment options include standard autologous stem cell transplantation as the standard of care for young patients with newly diagnosed MM. The arrival of novel immunomodulatory drugs, proteasome inhibitors, and monoclonal antibodies have inarguably been essential in induction, maintenance, and possibly consolidation treatment options for MM patients [15]. Unfortunately, for many MM patients, the occurrence of significant morbidity associated with end-organ destruction and potential direct cardiovascular complications secondary to the use of proteasome inhibitors and immunomodulatory drugs can occur [15].

Since the presence of fQRS has not been previously described in MM, we decided to conduct this pilot analysis as we practice at an institution that has a MM cancer center. Our aim was to determine the prevalence of fQRS among an unselected group of MM patients at different stages of their treatments.

## Materials and methods

For this pilot study, we randomly selected a group of MM patients referred for an ECG. These tracings were obtained during routine visits as part of their regular treatments or evaluation as dictated by protocols used at the MM Cancer Center at the University of Arkansas for Medical Sciences (UAMS), Little Rock, AR. 

The University of Arkansas for Medical Sciences Institutional Review Board approved this retrospective study and did not require a signed consent form to proceed with data collection. 

From November 2019 to July 2020, we identified all MM patients that were referred to our ECG laboratory at University of Arkansas for Medical Sciences for a 12 lead ECG tracing. All ECGs were obtained during these routine visits, prompted as part of each individual patient protocol. As originally intended as part of this pilot study, we purposely wanted to capture MM patients at different stages of their treatment to comment on the overall prevalence of fQRS. All ECGs were collected from a MUSE (GE Healthcare, Chicago, Illinois) ECG reading station.

Traditional ECG recordings are routinely used to detect fQRS. No specific setting is required as the current set standard high-pass filter: 0.05-20 Hz (usually 0.15 Hz), low-pass filter: 100-150 Hz, AC filter: 50 or 60 Hz, paper speed: 25-50 mm/sec (usually 25mm/sec) and voltage: 1mm/mV are the set settings currently used in routine 12-lead ECG recording [4-6].

Even when a low-pass filter could be used to reduce electrical and musculature noises when recording the 12-lead ECG, the use of these low-pass filters influence the detection of fQRS [16]. Therefore, in regular clinic ECG acquisition, as in the case for this study analysis, our MUSE ECG reading station uses a standard frequency of 150 Hz to acquire and record all ECG tracings. For our study, we purposely only used the standard, routinely used, high-frequency filters to report prevalence of the fQRS in MM patients. We did not analyze if changes in fQRS signals would occur when switching to low-filter settings. 

For this study, we followed Das et al. previously defined criteria for recognition of fQRS [4-6]. Specifically, fQRS will be identified if an additional R wave (R') or notching in the nadir of the R wave or the S wave, or the presence of >1 R' (fragmentation) in two contiguous leads were identified from the surface 12-lead ECG on QRS complexes. As previously, suggested either left or right bundle branch block patterns (QRS ≥ 120 ms), as well as incomplete bundle patterns, were excluded from our analysis [4-6].

Continuous variables are presented as mean ± SD, and categorical variables are presented as n of patients (%). Continuous data were analyzed using Student's t-test. Categorical data were compared using the χ2-test or Fisher's exact test. A p-value < 0.05 was considered statistically significant. 

## Results

During the stipulated study period (November 2019 to July 2020), 170 MM patients were seen at the University of Arkansas for Medical Sciences. Of these, 137 MM patients had an ECG. A total of three of these patients were excluded due to an interventricular conduction defect. 

Therefore, our study population consisted of 134 patients (age 61 ± 12 years, 74 males) at different stages of their treatment. All ECGs were collected from a MUSE ECG reading station.

A total of 104 patients were in sinus rhythm at the time when the ECG was acquired; 16 patients had sinus bradycardia, 13 had sinus tachycardia, and one had atrial fibrillation. 

Using the Das et al criteria, we identified fQRS in 56 MM patients (42%) [4-6].

Given the previously described gender differences, we then examined if such gender distinction was also seen in our MM group. In our study population, fQRS was more common among MM men (46%, mean age 61 ± 10 years) than MM women (37%, mean age 61 ± 13 years). 

To account for the possible disparity in left ventricular mass that is known to occur between males and females, we used the total amplitude of the R wave in the precordial lead V5, a previously described surrogate marker [1]. Based on this analysis, we found no statistical difference between males and females with regards to lead V5 R amplitude (1.3 ± 0.5 versus 1.3 ± 0.5 mV, p=NS) that could explain the changes seen in terms of fQRS.

**Figure 1:**
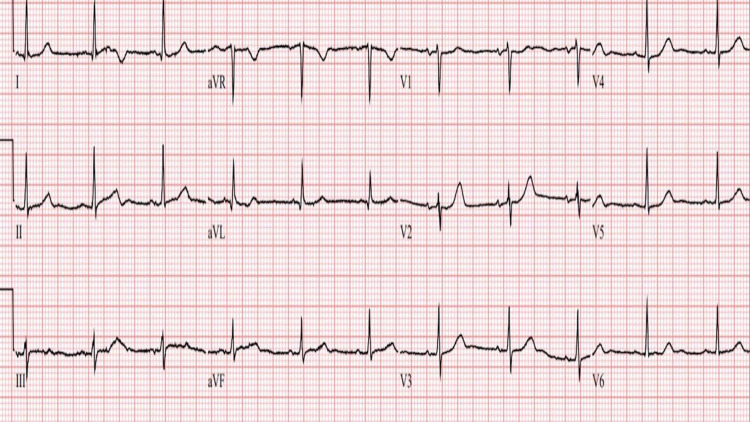
Normal ECG from a MM patient A representative 12 lead ECG tracing from a multiple myeloma (MM) patient without fQRS. Therefore, a normal ECG without depolarization abnormalities.

**Figure 2:**
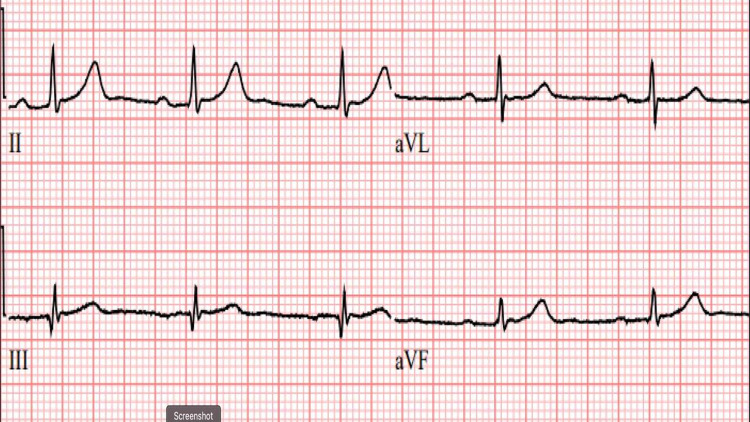
Zoom image showing a normal QRS configuration Zoom image of a representative ECG tracing without fQRS from a different multiple myeloma (MM) patient without these depolarization abnormalities.

**Figure 3:**
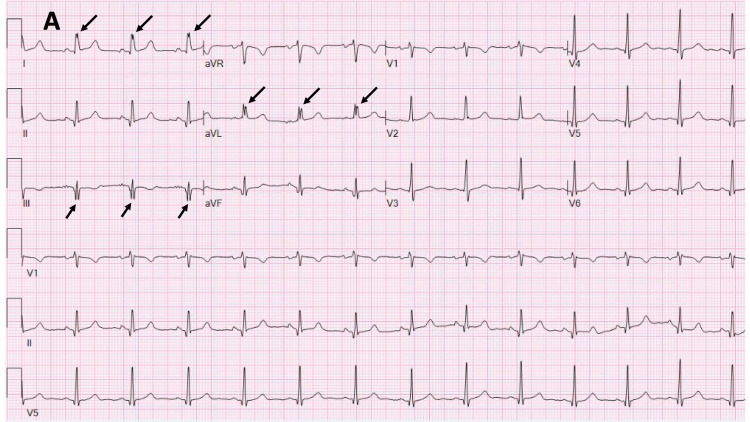
Abnormal fQRS from a MM patient Representative 12 lead ECG tracing from a multiple myeloma (MM) patient showing fQRS in leads I, aVL, and lead III.

**Figure 4:**
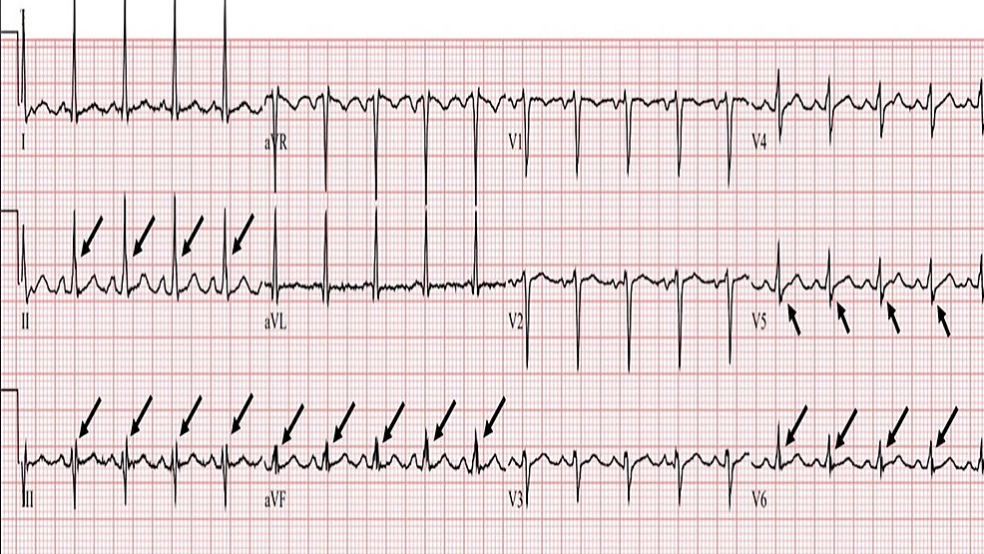
A second abnormal ECG showing fQRS from a MM patient Representative 12 lead ECG tracing from a different multiple myeloma (MM) patient showing fQRS in leads II, III, aVF, and leads V5 and V6.

**Figure 5:**
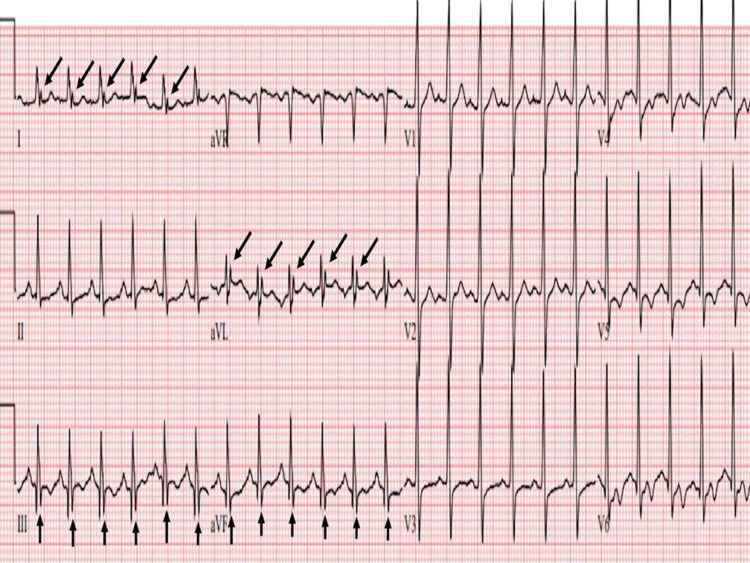
Third abnormal ECG showing fQRS from a different MM patient Representative 12 lead ECG tracing from another multiple myeloma (MM) patient also showing fQRS in leads I, aVL, and leads III and aVF.

**Figure 6:**
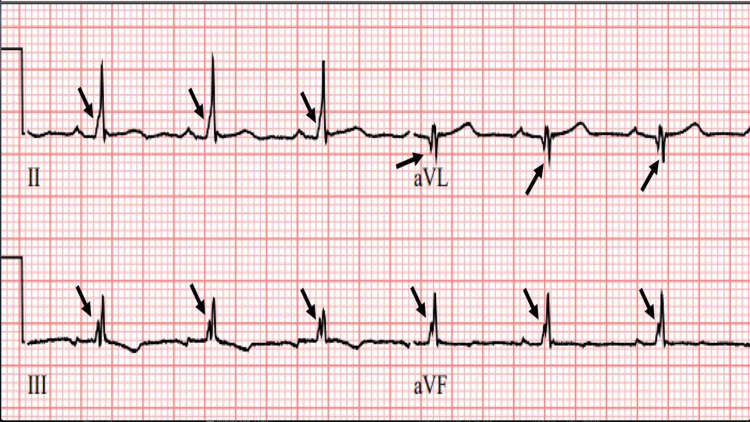
Zoom ECG from a different MM patient showing fQRS Zoomed ECG from a multiple myeloma (MM) patient showing fQRS only in limb leads II, III, aVF, and aVL.

**Figure 7:**
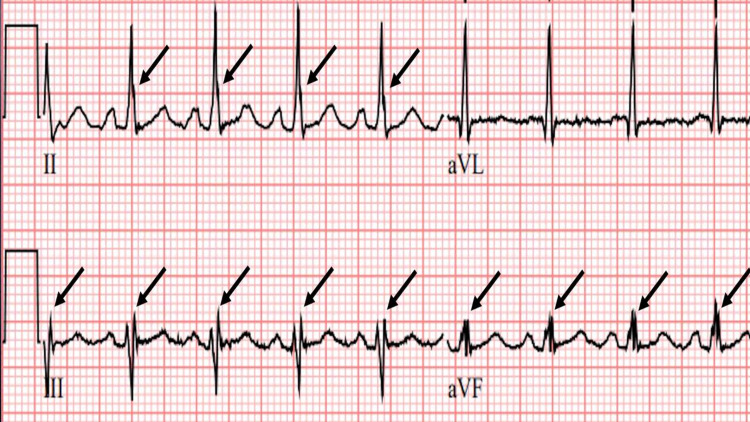
Different zoom ECG tracing showing abnormal fQRS from a MM patient Zoomed ECG from a different multiple myeloma (MM) patient showing fQRS only in limb leads II, III, and aVF.

**Figure 8:**
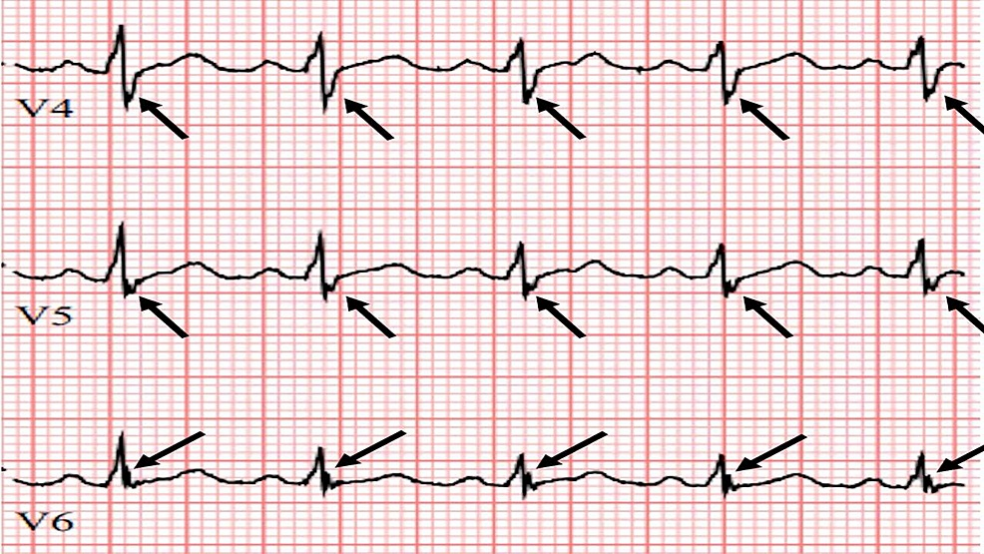
Abnormal zoom precordial fQRS from a different MM patient Zoomed ECG from another multiple myeloma (MM) patient showing fQRS abnormalities only in precordial leads V4, V5, and V6.

Based on the previously mentioned Das et al.'s definition [4-6], we would like to highlight the differences seen in terms of a normal QRS configuration (without fQRS) obtained from two different MM patients, as seen in Figures 1 and 2. This contrast when fQRS is noted, as seen in three different MM patients (Figures 3-5). Furthermore, we also want to showcase fQRS these depolarization abnormalities by providing zoom views of these QRS abnormalities on different leads, as shown in Figures 6-8.

## Discussion

To our knowledge, we have documented for the first time, based on our pilot analysis, the prevalence of fQRS (42%) among an unselected population of MM patients, at different stages of their treatment. In addition, although fQRS is mostly seen in MM male patients (46% versus 37%), the prevalence observed of fQRS among MM female patients from surface ECGs far exceeds that seen in either gender from the general population [2].

As survival rates continue to improve among cancer patients, it has become imperative to find noninvasive ways to identify which of these cancer survivors exposed to current treatment options, that now include newer chemotherapeutic and immunotherapeutic agents, might develop subclinical cardiac toxicity, particularly when the latter has been mostly associated to the development of myocardial fibrosis [16]. Consequently, early identification of cardiotoxicity should be at the forefront of surveillance protocols among cancer survivors. Even when we still do not have clear evidence guidelines on how to guide our approach to monitor for cardiovascular toxicity across the board of all cancer patients [17]; it is well-known that this approach should be at least in principle used more attentively in patients treated with anthracyclines [18].

To that effect, not only is early identification of myocardial injury is critical but also, mitigation of the effects of left ventricular dysfunction induced by cancer therapy have been one of the main goals of cardiotoxic investigation. 

Traditionally the use of multigated acquisition scans or radionuclide angiography, basic and advanced echocardiography as well as cardiac magnetic resonance imaging have been at the forefront of these noninvasive techniques for the detection of cardiotoxicity [18-20]. These techniques have either used a drop in left ventricular systolic function or incorporated different imaging algorithms of tissue characterization to detect subclinical cardiotoxic effects of cancer therapy [21, 22].

With increasing health care costs, the search for additional noninvasive markers has intensified. fQRS is one of such novel noninvasive markers, that have been suggested as a possible alternative. This depolarization abnormality, at least in other clinical settings, has been associated with myocardial fibrosis [1-8].

More importantly and based on a mechanistic point of view, identification of fQRS in breast cancer [10,11] and non-Hodgkin lymphoma patients treated with R-CHOP regimens [12] were the drivers that prompted our interest in conducting this pilot analysis among an unselected group of MM patients, at different stages of their treatment, particularly when the incidence of MM has continued to increase based on findings recently published from studies assessing the global burden of MM since 1990 [23]. Furthermore, the undeniable reality that MM is occurring in younger patients (median age at diagnosis 70 years old [14,23,24] is critically important as MM clinical outcomes, not only are these dependent on each individual patient's overall fitness and underlying health status but also, age with a recognized cut off < 65 years, is a good prognostic marker [25]. Consequently, identifying noninvasive alternatives to recognize subclinical cardiotoxic effects of therapy is crucial, particularly when close follow-up of MM has become the norm given the significant morbidity due to its end-organ damage infiltration and destruction [15] as well as the development of adverse cardiovascular events. Specifically, MM is known to have both direct and indirect effects and may cause or aggravate hypertension, ischemic heart disease, congestive heart failure, arrhythmias, or result in venous as well as arterial thromboembolism, arterial thromboembolism, or pulmonary hypertension [25,26]. More importantly and quite relevant to our claim that MM, in fact, is associated with myocardial fibrosis, and fQRS could be useful in identifying these patients. The detrimental effect of MM therapy was first reported after a 60-year-old MM female developed heart failure associated with a reduction in left ventricular systolic function following initiation of bortezomib. Cardiotoxicity was documented as midwall hyperenhancement consistent with midwall fibrosis using gadolinium enhanced cardiovascular magnetic resonance scan imaging [27]. In addition, the cardiotoxic effects of carfilzomib were documented using global longitudinal strain imaging in a 60-year-old MM female patient after she developed severe congestive heart failure with elevated cardiac biomarkers and reduction in left ventricular systolic function [28].

Despite our claim to be the first to ever document fQRS in MM patients, we do acknowledge the following study limitations. First, the small sample size. Despite being small, most cancer-based studies do not comprise large-scale numbers; moreover, this was a pilot study. Second, we did not include a control group. The main goal was to assess prevalence in MM patients, as prevalence in the general population has already been described by other studies, cited for reference. Finally, there might be those that might argue that we did not provide the date/time of bone marrow transplant and prior chemotherapy/immunomodulatory treatments in our patient population. In addition, lack of availability with regards to prior cardiac testing data in those patients referred to our institution solely for MM care, limited our ability to assess if prior hypertension of coronary artery disease were relevant issues. 

We purposedly conducted our pilot study as originally intended to simply get a glimpse of the overall prevalence of fQRS in MM while avoiding the confounding effect of different treatments given the small number of patients enrolled in the analysis. Not only did we note considerable variability in treatment combination and duration of therapy prior to arrival at our center, but also, the advent of so many new therapies over the last five years complicates the interpretation of our results. To solve this limitation, a larger number of patients is needed to power our analysis. Therefore, we eliminated these limitations by simply using MM diagnosis as entry criteria. The indication for ECG on our study patients was mainly driven by the institution's cancer care treatment protocols. However, our main study aim, as stated prior to initiation of the study, was to simply determine the prevalence of fQRS among an unselected group of MM patients at different stages of their treatments. Based on these results additional prospective studies can then be carried out. 

The importance of our results now goes beyond the possibility of proposing that cardiac fibrosis, as stated in our discussion, might be ultimately related to the development of arrhythmias and heart failure in MM patients, but most importantly, determine what the relationship that might exist between fQRS and cardiac amyloid is. Between 12 and 15% of MM patients have symptomatic amyloid light-chain (AL) amyloidosis, and up to 38% of newly diagnosed MM patients might have clinically occult AL amyloidosis [29]. Therefore, it is imperative that large multicenter-prospective, collaborative studies should then be conducted not only using ECG and cardiac imaging but also cardiac biomarkers as well as MM markers to determine how fQRS can be useful in identifying the presence and extent of cardiac fibrosis and AL amyloid with overall clinical outcomes of MM patients.

As a final note, it is important to comment on the overall potential diagnostic value of the fQRS complex in terms of myocardial scar detection if additional studies are to be conducted. In a systematic review and meta-analysis of the literature performed by Sadeghi and associates, the presence of fQRS was more sensitive; however, less specific than Q wave on the surface ECG to identify myocardial scar. They specifically, reported that a combination of fQRS with Q wave in a 12-lead ECG results in up to 74% sensitivity and 92% specificity [30]. We did not include Q wave analysis in our study since the original studies reported by Das et al. made no mention of the Q wave when reporting fQRS [4-6].

## Conclusions

To our knowledge, this is the first study describing the prevalence of fQRS in MM patients. Certainly, the next logical step is to first follow MM patients early on from diagnosis and all throughout their different treatment sessions to determine the onset and evolution of fQRS. The presence of fQRS should now be correlated with not only recognized subclinical markers of cardiotoxicity but also with cardiac imaging tools so that specific correlations between fQRS and myocardial fibrosis and/or left ventricular function could be made. Finally, hard-end outcomes should be envisioned as the final point on determining the potential utility of fQRS when following MM.

Surely, due to the time of data acquisition, there might be those that might argue what impact, if any, the ongoing COVID-19 pandemic might have had on fQRS. Unfortunately, as with most COVID-19 data, we are in no position to make any comment at this time. However, these ECG tracings were obtained in outpatients at the time of their scheduled routine visits, and COVID surveillance protocols were in place, particularly in this immunocompromised patient group.

We can only conclude that based on this random sampling analysis, fQRS appears highly prevalent on surface ECG in patients with MM, but additional prospective data and complimentary testing is required to draw any conclusions of the potential utility of this ECG finding.
